# Preparation of *Trichoderma asperellum* Microcapsules and Biocontrol of Cucumber Powdery Mildew

**DOI:** 10.1128/spectrum.05084-22

**Published:** 2023-04-27

**Authors:** Qi Qi, Chengcheng Fan, Hongqu Wu, Lili Sun, Chuanwang Cao

**Affiliations:** a Key Laboratory of Sustainable Forest Ecosystem Management-Ministry of Education, Northeast Forestry University, Harbin, People’s Republic of China; b Hubei Biopesticide Engineering Research Center, Wuhan, Hubei, People’s Republic of China; Agroscope

**Keywords:** *Trichoderma asperellum* microcapsules, sodium alginate, cucumber powdery mildew

## Abstract

Microencapsulation is an important technique for protecting the viability and activity of microorganisms under adverse environmental conditions. To improve biological control, controlled-release microcapsules of *Trichoderma asperellum* were prepared and embedded in combinations of the biodegradable wall materials sodium alginate (SA). The microcapsules were evaluated for their ability to control cucumber powdery mildew in the greenhouse. The results showed that the highest encapsulation efficiency of 95% was obtained by applying 1% SA and 4% calcium chloride. The microcapsules provided good, controlled release and UV resistance, and could be stored for a long time. The greenhouse experiment revealed that the *T. asperellum* microcapsules had a maximal biocontrol efficiency of 76% against cucumber powdery mildew. In summary, embedding *T. asperellum* in microcapsules is a promising technique to improve the survivability of *T. asperellum* conidia. The *T. asperellum* microcapsules exerted significant biocontrol efficiency against cucumber powdery mildew.

**IMPORTANCE** *Trichoderma asperellum* is widely found in plant roots and soil and has been used for the biocontrol of various plant pathogens; however, the control efficiency of *T. asperellum* is usually unstable in field trials. To improve the control efficiency of *T. asperellum*, in the present study, *T. asperellum* microcapsules were prepared using sodium alginate as wall material to reduce the effects of temperature, UV irradiation, and other environmental factors on its activity, and to significantly improve its biocontrol efficiency on cucumber powdery mildew. Microcapsules can prolong the shelf life of microbial pesticides. This study provides a new way to prepare a biocontrol agent against cucumber powdery mildew with high efficiency.

## INTRODUCTION

*Trichoderma asperellum* is widely found in plant roots and soil. Research has confirmed that *Trichoderma* spp. has various mechanisms of antagonism, antibiosis, competition for space and nutrients, induction of systemic resistance in plant, and mycoparasitism to achieve biological control (1). However, *Trichoderma* is susceptible to environmental changes, such as pH, UV light, and temperature, showing low efficiency to control agricultural and forestry diseases (2–5). Therefore, employing different forms of delivery could improve the viability of conidia (6, 7). For instance, microencapsulated conidia of Metarhizium *anisopliae* using sodium alginate (SA) showed that the conidia activity was 80% after storage at 4°C for 6 months, while that of the bare conidia was less than 50% under identical conditions (8–10).

Microencapsulation can improve the viability and stability of biocontrol strains and is harmless to the environment (11). The extrusion method is usually used to make microcapsules because of its low cost, mild conditions, simple operation, and short preparation time (12). To date, many biocontrol microorganisms have been prepared into microcapsules by extrusion methods, such as Bifidobacterium longum (13), Bacillus velezensis NH-1 (14), and Pseudomonas putida Rs-198 (15).

Alginate, a natural copolymer, has attracted a lot of attention in the food, medical, and agriculture fields because of its biocompatibility, low cost, low toxicity, and mild preparation conditions (by addition of divalent cations such as Ca^2+^) (16). In the traditional extrusion generation technique, the SA solution is dripped through a syringe needle or nozzle into a CaCl_2_ solution to form spherical and uniform calcium alginate hydrogel beads. Compared with traditional methods, such as emulsification and spray drying, the microcapsule preparation conditions using an encapsulator are mild, with simple operation and a short preparation time. Microcapsules are the most popular formulation because of their uniform shape, small size, and good encapsulation properties, leading to relatively high viability of the encapsulated microorganisms (12).

Cucumber powdery mildew, caused by *Sphaerotheca fuliginea*, is one of the severe diseases affecting cucumber production worldwide. Cucumber powdery mildew results in significant cucumber yield reduction, leading to huge economic losses (17–19). Although *Trichoderma* can control cucumber powdery mildew, its environmental stability must be improved. The objectives of the present study were to investigate SA for the microencapsulation of *T. asperellum* using the extrusion method to obtain various microcapsules and to then compare them in terms of their stabilization effect on the survival and release behavior of the encapsulated fungal conidia. In addition, the effects of the encapsulated fungus conidia on the reduction of cucumber powdery mildew incidence were investigated.

## RESULTS

### Biocompatibility of SA on *T. asperellum*.

The effects of different concentrations of SA on *T. asperellum* growth are shown in Table 1. The number of *T. asperellum* cells did not decrease observably in the medium with addition of SA compared to that in the control. When 1% alginate was added, *T. asperellum* still grew better and the conidia number was higher than those in the control (without SA). The results suggested that SA can be used as the wall material for *T. asperellum* microcapsules.

**TABLE 1:** Effects of sodium alginate concentration on conidial germination and growth rate of *T. asperellum*^*a*^

Sodium alginate concn (%)	Colony (colonies/plate)	Diam of colony/(mm·d^−1^)
0	52 ± 1.73a	17.17 ± 0.10ab
0.5	51 ± 0.88a	16.28 ± 0.15b
1.0	54 ± 2.33a	17.67 ± 0.38a
1.5	55 ± 1.15a	16.50 ± 0.63ab
2.0	55 ± 1.00a	17.00 ± 0.19ab

a Different lowercase letters in the same column indicate significant difference between treatments with different sodium alginate concentration by Duncan's multiple-comparison test (*P *< 0.05). Data are means ± SE (*n *= 3).

### Screening of the optimum conditions for microcapsule preparation.

**FIG 1:**
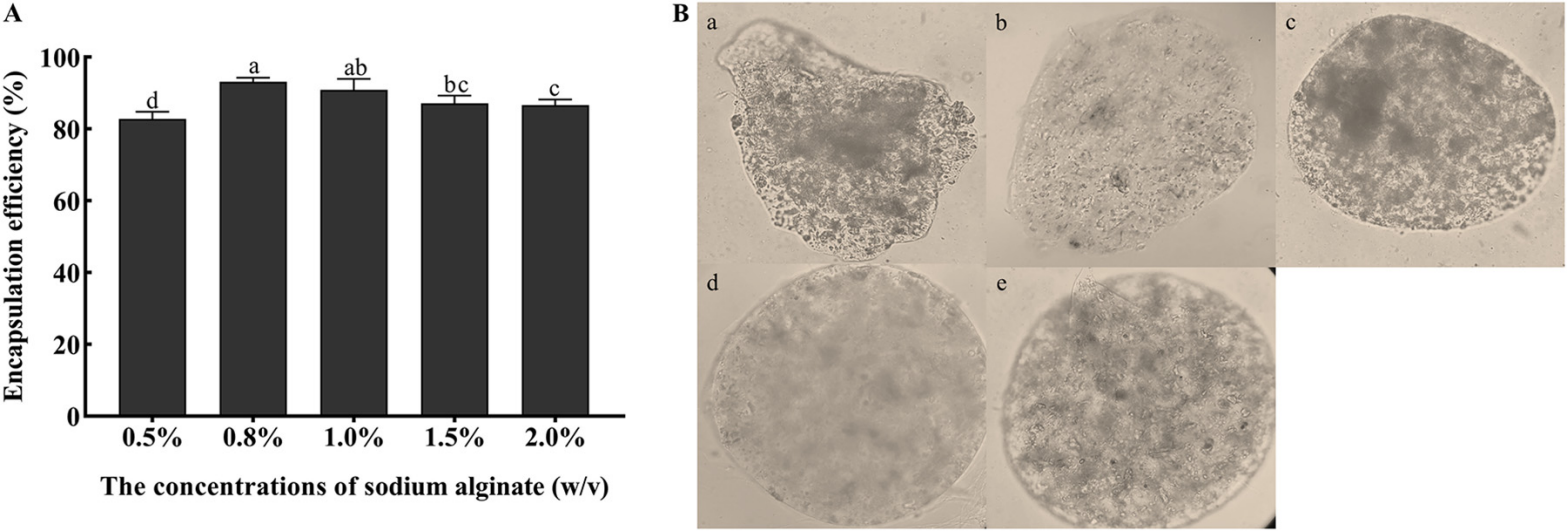
Effects of different concentrations of sodium alginate (SA) concentration on encapsulation efficiency and morphology of microcapsule. (A) Encapsulation efficiencies using different concentrations of SA with 2% CaCl_2_, a 1:1 ratio of conidia to SA, 60% stirring speeds, and curing for 10 min. Statistical significance among different treatments was calculated using one-way ANOVA, followed by Duncan's multiple-comparison test. Lower case letters show significant differences between treatments. (B) Microcapsule morphology using different concentrations of SA under an optical microscope (×400). (a) 0.5% SA, (b) 0.8% SA, (c) 1% SA, (d) 1.5% SA, (e) 2% SA.

The encapsulation efficiencies of the microcapsules prepared using different concentrations of SA were significantly different (Fig. 1A). With the increase in SA concentration, the encapsulation efficiency first increased and then decreased. Among them, the microcapsules prepared with 0.8% SA showed the fullest morphology, with 93% encapsulation efficiency. When the SA concentration was above 0.8%, the microcapsules formed clumps in the calcium chloride solution, with high mixture viscosity and low encapsulation efficiency (Fig. 1B). Therefore, 0.8% SA was the optimum concentration to prepare the microcapsules.

**FIG 2:**
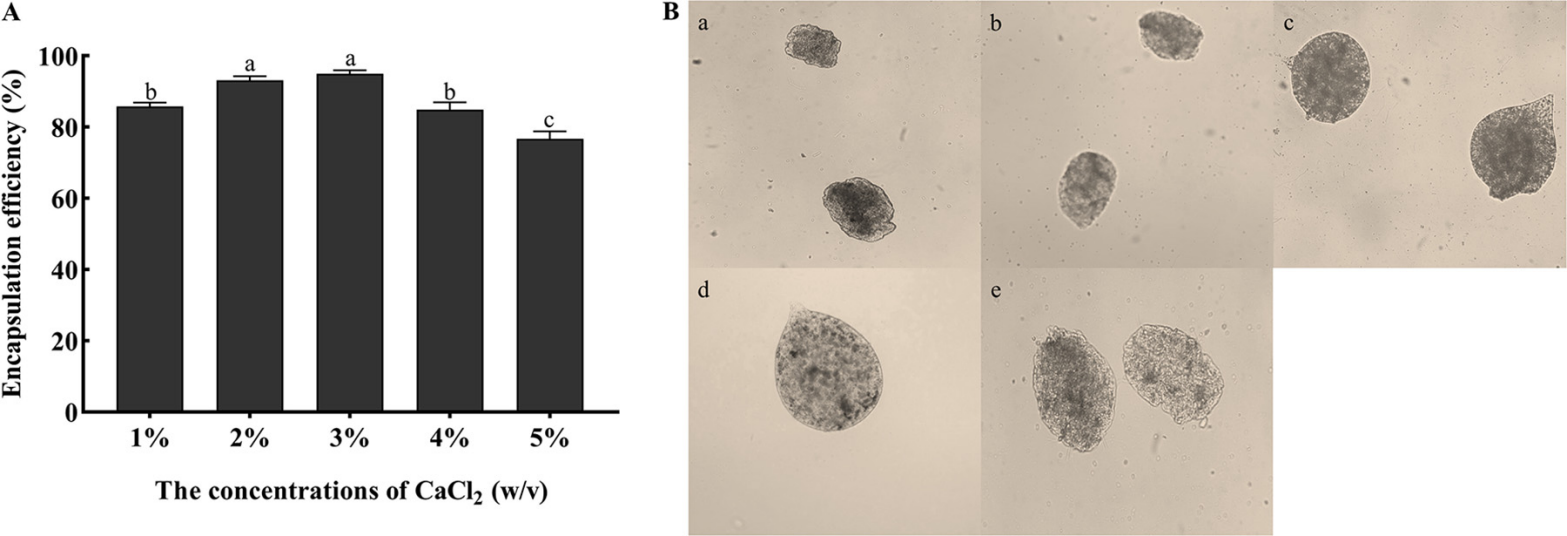
Effects of different CaCl_2_ concentration on encapsulation efficiency and morphology of microcapsule. (A) Encapsulation efficiencies using different concentrations of CaCl_2_ with 1% SA, a 1:1 ratio of conidia to SA, 60% stirring speeds, and curing for 10 min. Statistical significance among different treatments was calculated using one-way ANOVA, followed by Duncan's multiple-comparison test. Lower case letters show significant differences between treatments. (B) Microcapsule morphology using different concentrations of CaCl_2_ under the optical microscope (×100). (a) 1% CaCl_2_, (b) 2% CaCl_2_, (c) 3% CaCl_2_, (d) 4% CaCl_2_, (e) 5% CaCl_2_.

As the concentration of CaCl_2_ increased, SA and Ca^2+^ bound on the surface of the microcapsules to form a denser protective layer, which could effectively wrap the conidia inside the microcapsules. The highest encapsulation efficiency was 95% when the concentration of CaCl_2_ was 3% (Fig. 2A). However, the encapsulation efficiency showed a downward trend when the concentration of CaCl_2_ was above 3%. When the concentration of calcium chloride was 5%, the encapsulation efficiency decreased to 77% because the high concentration of CaCl_2_ accelerated the cross-linking reaction of the microcapsules, leading to the formation of irregular microcapsules (Fig. 2B).

**FIG 3:**
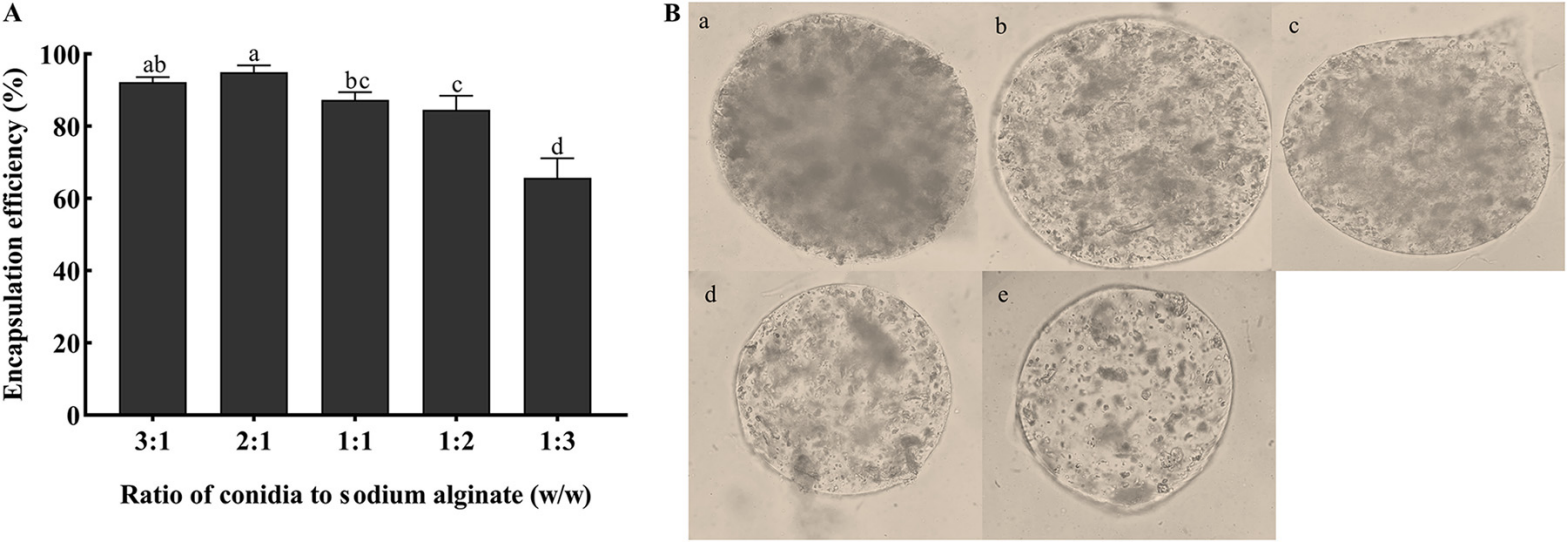
Effects of different ratios of conidia to SA on encapsulation efficiency and morphology of microcapsule. (A) Encapsulation efficiencies using different ratios of conidia to SA with 1% SA, 2% CaCl_2_, 60% stirring speeds, and curing for 10 min. Statistical significance among different treatments was calculated using one-way ANOVA, followed by Duncan's multiple-comparison test. Lower case letter show significant differences between treatments. (B) Microcapsule morphology using different ratios of conidia to SA under the optical microscope (×400). (a) 3:1, (b) 2:1, (c) 1:1, (d) 1:2, (e) 1:3.

The encapsulation efficiency of the microcapsules was 95% when the ratio of conidia to SA was 2:1 (Fig. 3A). When the ratio of conidia to SA was increased to 3:1, the encapsulation efficiency decreased to 92% because of the lower stability of microcapsules. When the proportion of SA was too high, the encapsulation efficiency decreased. Moreover, the wall of microcapsules was thicker, and the solution became too viscous, leading to irregularly formed microcapsules (Fig. 3B). When the ratio of conidia to SA was 1:3, the encapsulation efficiency decreased to 66%.

**FIG 4:**
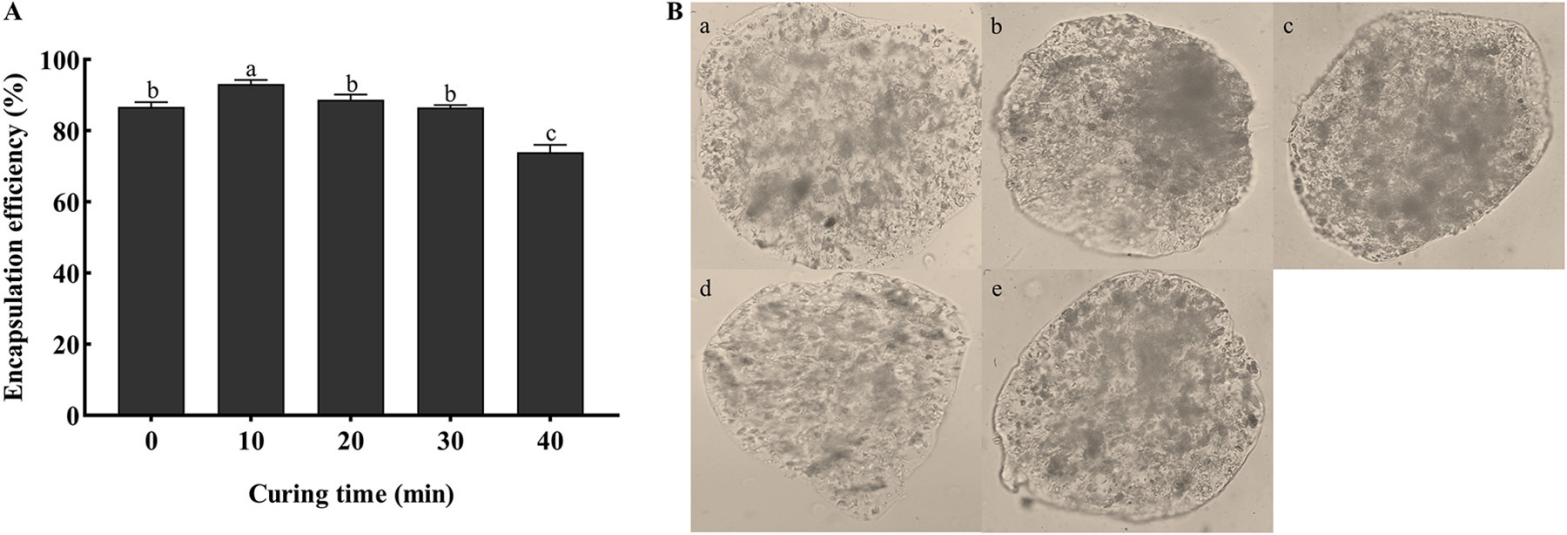
Effects of different curing times on encapsulation efficiency and morphology of microcapsule. (A) Encapsulation efficiencies using different curing times with 1% SA, a 1:1 ratio of conidia to SA, 2% CaCl_2_, and 60% stirring speeds. Statistical significance among different treatments was calculated using one-way ANOVA, followed by Duncan's multiple-comparison test. Lower case letters show significant differences between treatments. (B) Microcapsule morphology using different curing times under the optical microscope (×400). (a) 0 min, (b) 10 min, (c) 20 min, (d) 30 min, (e) 40 min.

The highest encapsulation efficiency was 93% after curing for 10 min (Fig. 4A). The encapsulation efficiency was 87% at 0 min, and the wall hardness was low and unstable because the curing time was too short (Fig. 4B). The encapsulation efficiency was 74% after curing for 40 min; however, a longer curing time could destroy the capsule wall structure, change the microcapsule density, and decrease the encapsulation efficiency.

**FIG 5:**
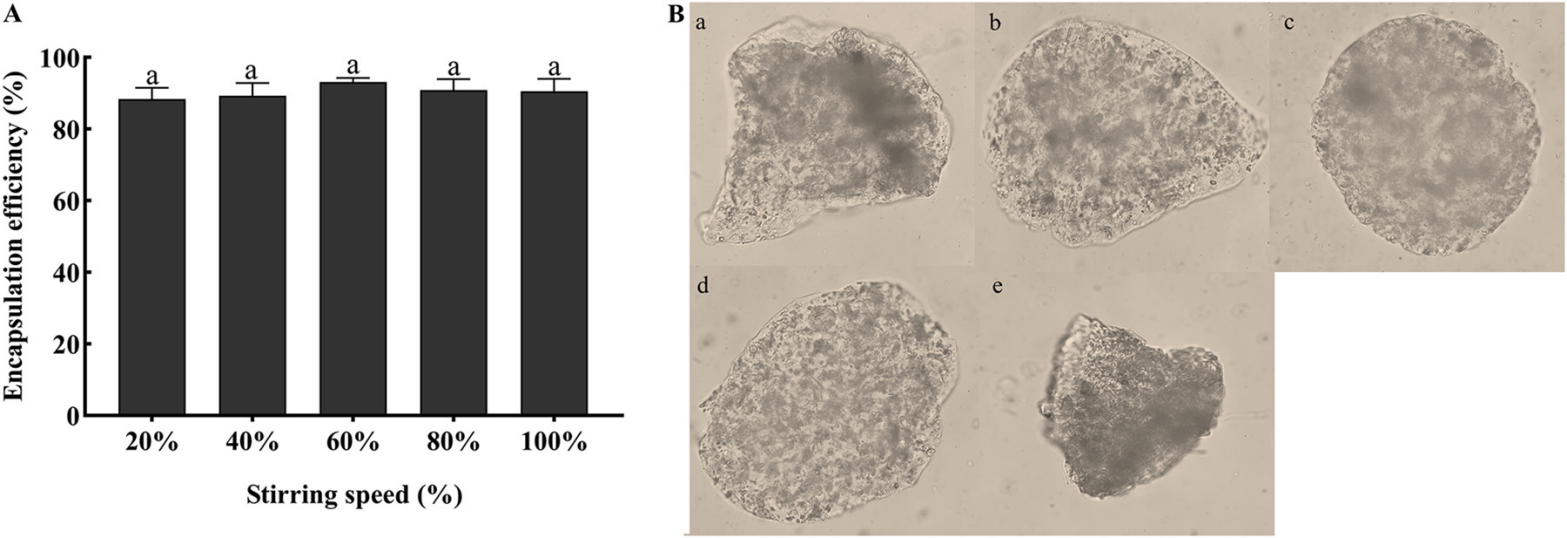
Effects of different curing times on encapsulation efficiency and morphology of microcapsule. (A) Encapsulation efficiencies using different stirring speeds with 1% SA, a 1:1 ratio of conidia to SA, 2% CaCl_2_, and curing for 10 min. Statistical significance among different treatments was calculated using one-way ANOVA, followed by Duncan's multiple-comparison test. Lower case letters show significant differences between treatments. (B) Microcapsule morphology using different stirring speeds under the optical microscope (×400). (a) 20%, (b) 40%, (c) 60%, (d) 80%, (e) 100%.

The encapsulation efficiency at different stirring speeds was above 88% but was not significantly different among the groups (Fig. 5A). However, different stirring speeds affected the shape of the microcapsules (Fig. 5B). A slower stirring speed was not conducive to droplet dispersion. The formed microcapsules were irregular in a faster stirring speed.

### Orthogonal optimization of the formulation.

**FIG 6:**
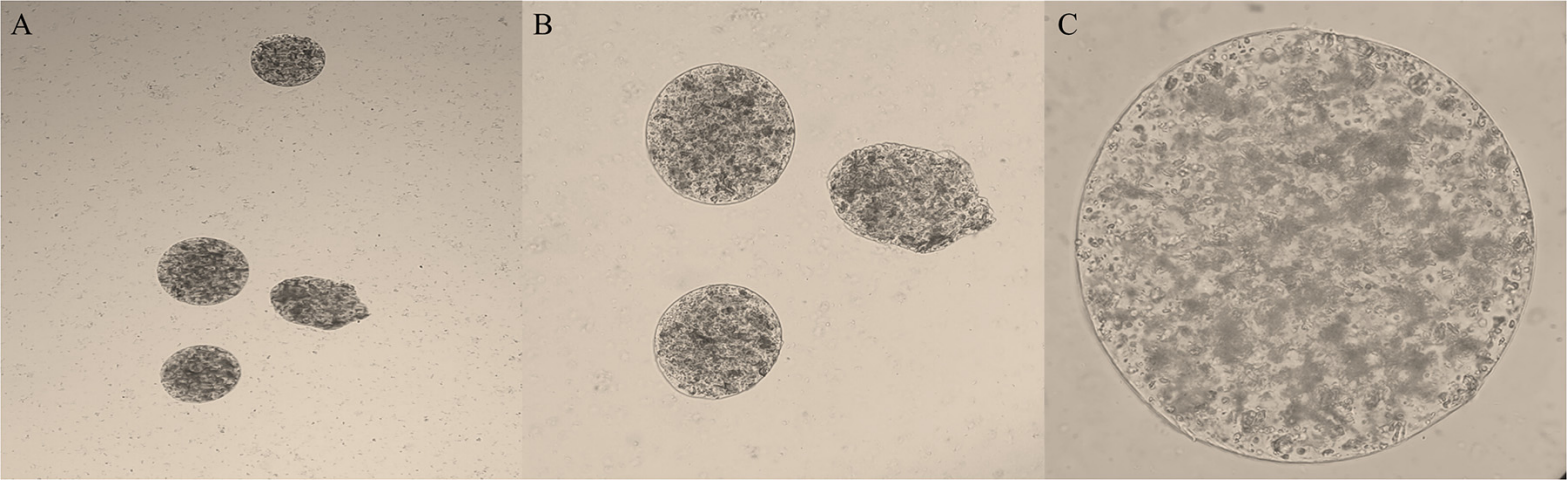
Morphology of the optimal formulation under a light microscope. (A) ×40, (B) ×100, and (C) ×400.

In this study, an L_9_ (3^4^) orthogonal test was used to analyze the effect of four factors on encapsulation efficiency. The L_9_ (3^4^) orthogonal test along with the experimental results and range analysis are presented in Table 2. The optimum result was A_3_B_2_C_3_D_3_, and optimum conditions were obtained as follows: SA concentration 1% (wt/vol), CaCl_2_ concentration 4% (wt/vol), 2:1 (wt/wt) of conidia to SA, and curing for 15 min, respectively. Under these conditions, the *T. asperellum* microcapsules were obtained with 95% encapsulation efficiency and with the best microcapsule morphology (Fig. 6). This matrix shows that the influence on the encapsulation efficiency decreased in the order of A >B > D > C, according to the R values. A larger R value indicates a greater influence of this factor on the encapsulation efficiency. The results also showed that increasing SA concentration could also improve the encapsulation efficiency of the microcapsules significantly.

**TABLE 2:** Orthogonal design of optimization results

Treatments	Factors				Encapsulation efficiency /%
	A	B	C	D	
1	1	1	1	1	71
2	2	1	2	2	75
3	3	2	1	2	94
4	2	2	3	1	97
5	3	3	2	1	90
6	1	3	3	2	81
7	3	1	3	3	94
8	2	3	1	3	93
9	1	2	2	3	88
k_1_	79.9	80.0	86.0	85.9	
k_2_	88.3	93.0	84.6	83.5	
k_3_	93.1	88.1	90.6	91.8	
Range (R)	13.2	13.0	6.0	8.3	

### Characteristics of microcapsules.

**FIG 7:**
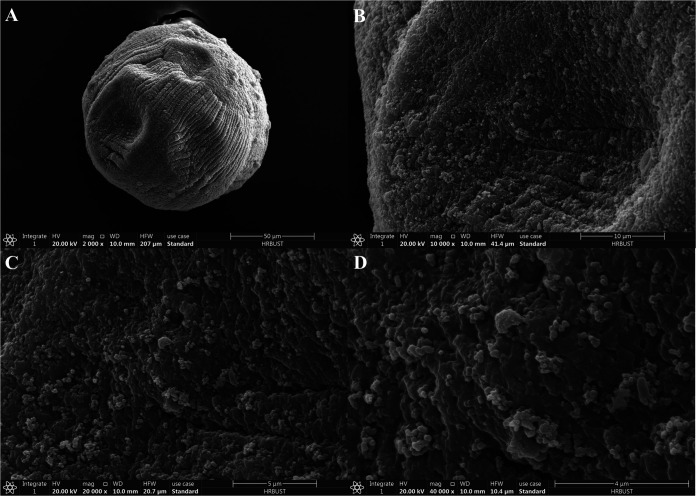
Observation of sodium alginate microcapsules using SEM. (A) ×2,000, (B) ×10,000, (C) ×20,000, and (D) ×40,000.

**(i) Scanning electron microscopy observations of the microcapsules.** The surface morphology of microcapsules was observed using a scanning electron microscope (Fig. 7). After drying and dehydration, the surface of the microcapsule was slightly depressed, and many cracks were formed. Further observation at higher magnification revealed that the surface of the microcapsule had many pores, which were conducive to the release of the conidia from inside the microcapsule.

**FIG 8:**
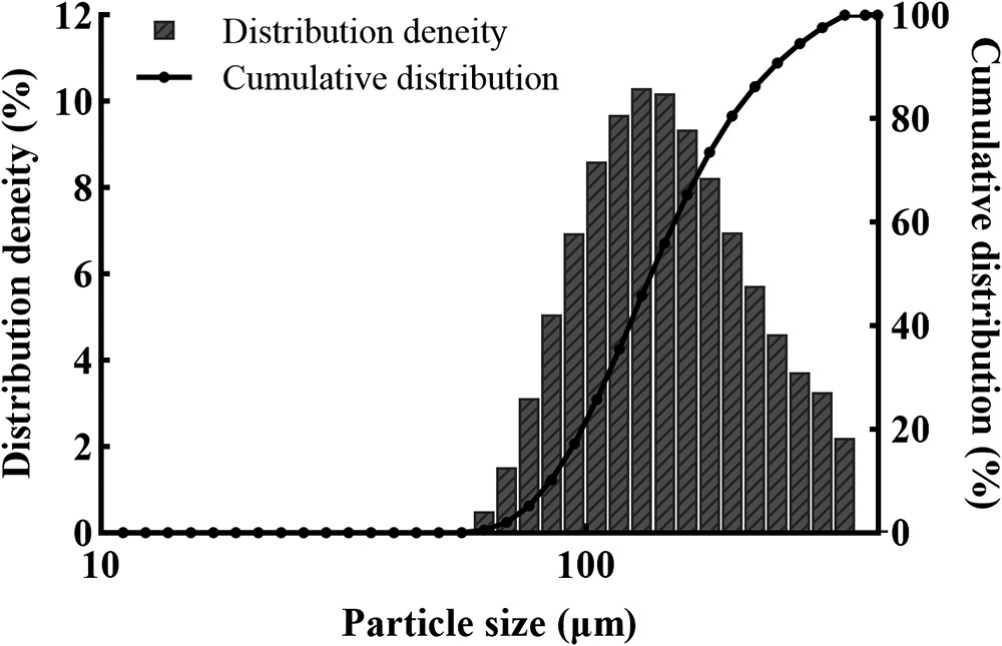
Particle size distribution of the microcapsules.

**(ii) Particle size.** The particle size was measured using a laser particle size distribution analyzer (shown in Fig. 8). The particle size distribution of the microspheres was expressed using the median diameter (D50) with 151.8 μm, and most of the microspheres were distributed in the range of 94.1 to 269.7 μm (D10–D90).

**FIG 9:**
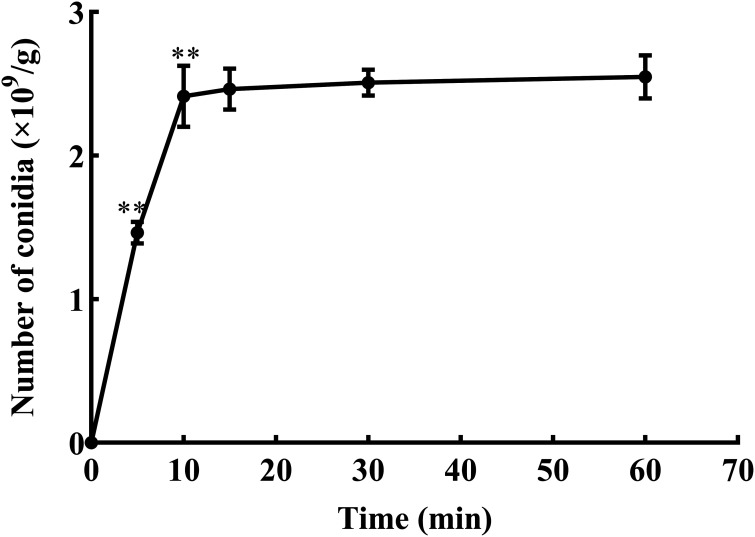
Release of encapsulated conidia in lysate. ** and * show significant differences between the treatments at two adjacent time points, as assessed using Student's *t* test at the 0.01 and 0.05 level, respectively.

**(iii) Loading capacity.** The experimental results showed that the loading capacity of the microcapsules was 2.53 × 10^9^/g under the optimal preparation conditions. The release profiles of conidia-loaded microcapsules were determined in lysis solution (Fig. 9). A total of 2.41 × 10^9^/g *T. asperellum* conidia could be released from 1 g of microcapsules within 10 min. All *T. asperellum* conidia embedded in the microcapsules could be released into the lysis solution within 15 min. The concentration of spores in the lysis solution was 2.51 × 10^9^/g at 30 min. The results showed that the microcapsules had the advantage of rapid release.

**(iv) Water content and swelling measurements.** Swelling is the most important factor that controls the release rate from microcapsules. When dispersed in deionized water, microcapsule swelling affects the release of the active agent. The swelling behavior of polymers depends on the properties of the polymer structure. The swelling rate of the microcapsules was 136% (Table 3). The water content of the microcapsules was 18% (Table 3).

**TABLE 3:** Determination of water content and swelling rate of microcapsules

Treatment	Wt before hot drying (mg)	Wt after hot drying (mg)	Avg water content (%)	Wt before swelling (mg)	Wt after swelling (mg)	Avg swelling rate (%)
1	103.0	83.0	18 ± 1.2	97.7	231.5	136 ± 6.4
2	71.9	60.4		77.2	190.0	
3	60.3	48.5		87.8	196.6	

**FIG 10:**
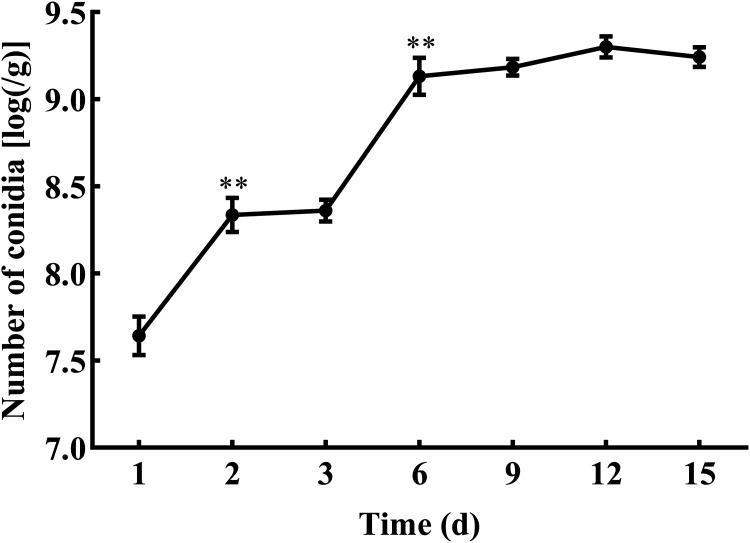
Release of encapsulated conidia in water for 15 days. ** and * show significant differences between the treatments at two adjacent time points, as assessed using Student's *t* test at the 0.01 and 0.05 level, respectively.

**(v) The release of conidia from microcapsules.** The numbers of conidia released from the microcapsules into sterile water were detected after 1, 2, 3, 6, 9, 12, and 15 days (Fig. 10). At 1 day, conidia were slowly released from microcapsules. After 3 days, the conidia were still slowly released into water; thereafter, the number of conidia released into water increased continuously along with the disintegration of the microcapsules. After 12 days, 80% of conidia had been released into water. After 15 days, 91% of the encapsulated conidia had been released from the microcapsules.

**FIG 11:**
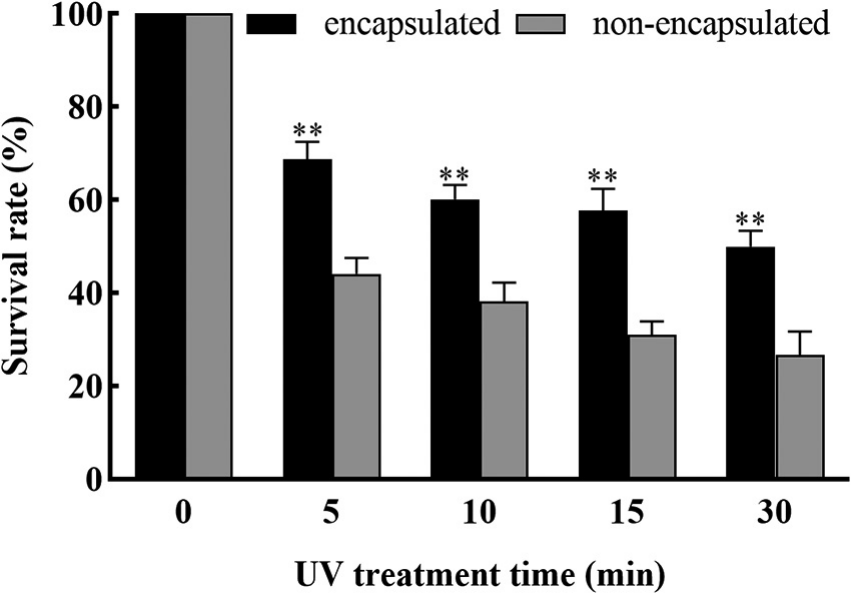
Survival rate of nonencapsulated and encapsulated conidia under UV treatment. ** and * show significant differences between the treatment and control groups, as assessed using Student's *t* test at the 0.01 and 0.05 level, respectively.

**(vi) Survival of conidia under UV irradiation.** The effects of UV irradiation on the survival of both nonencapsulated and encapsulated conidia are presented in Fig. 11. The number of viable nonencapsulated conidia declined rapidly and only 27% viable conidia could be detected after exposure to UV irradiation for 0.5 h. In contrast, the encapsulated conidia were relatively less sensitive to UV irradiation, and the conidia were still viable at 30 min, with a survival rate of 50%.

**FIG 12:**
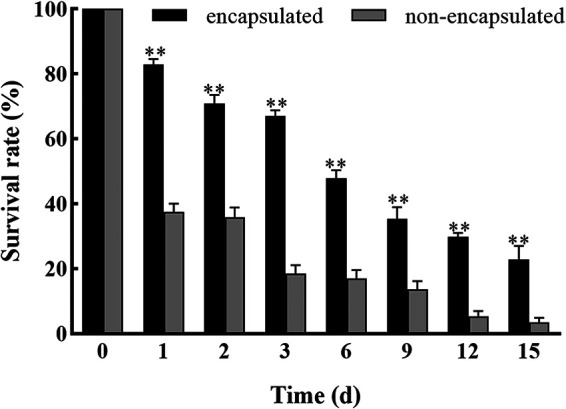
Survival rate of nonencapsulated and encapsulated conidia under heat treatments. ** and * show significant differences between the treatment and control groups, as assessed using Student's *t* test at the 0.01 and 0.05 level, respectively.

**FIG 13:**
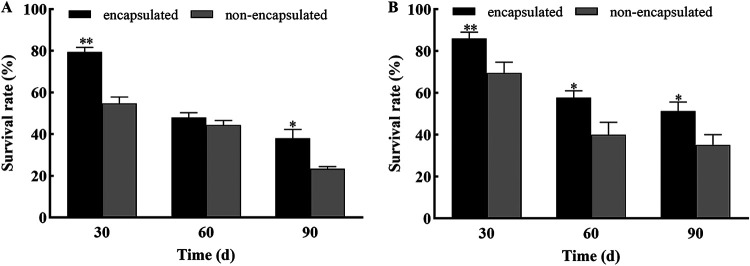
Survival rate of nonencapsulated and encapsulated conidia for 90 days. (A) Survival rate at room temperature. (B) Survival rate at 4°C. ** and * show significant differences between the treatment and control groups, as assessed using Student's *t* test at the 0.01 and 0.05 level, respectively.

**(vii) Storage stability.** The survival rate of the nonencapsulated samples decreased to 37% after treatment at 54°C for 1 d, while the survival rate of the conidia in the microcapsules was 23% after 15 days at 54°C (Fig. 12). The microcapsules were dried and stored for more than 3 months at room temperature and 4°C, respectively. The numbers of surviving conidia in the nonencapsulated samples or microcapsules were investigated at different time points (Fig. 13A). After 90 days, the survival rate of the conidia in the microcapsules was higher (38%) than that in nonencapsulated samples (23%) at room temperature. Moreover, the survival rate of the conidia in the microcapsules was higher (51%) than that in nonencapsulated samples (35%) at 4°C (Fig. 13B).

### Greenhouse experiment.

The control efficiency of the microcapsules on *S. fuliginea* was investigated using a greenhouse experiment (Table 4). The disease indexes of cucumber powdery mildew decreased after microcapsule treatment for 7 and 14 d. The disease indexes of the groups treated with all three concentrations of the microcapsules were significantly lower (2.46 to 3.41 and 4.09 to 5.86) than in the water control group (6.31 and 16.86) after 7 d and 14 d of application, respectively. At 7 days after *T. asperellum* microcapsule application (1.33 × 10^9^ CFU·g^−1^), the control efficiencies of the microcapsules on cucumber powdery mildew were 43%, 57%, and 61% at the dosages of 1,500 g·(hm^2^)^−1^, 3,000 g·(hm^2^)^−1^, and 7,500 g·(hm^2^)^−1^, respectively, while the control efficiency of the control B. subtilis wettable powder was 67%. After 14 days, the results showed that the control efficiencies of the microcapsules were 65%, 72%, and 76% at dosages of 1,500 g·(hm^2^)^−1^, 3,000 g·(hm^2^)^−1^, and 7,500 g·(hm^2^)^−1^, respectively, while the control efficiency agent B. subtilis wettable powder was 74%. According to the treatment with various concentrations of microcapsules, 7,500 g·(hm^2^)^−1^ had the best control efficiency, which was equivalent to that of the commonly used dose of the control agent.

**TABLE 4:** Control effects of *Trichoderma* microcapsule on cucumber powdery mildew^*a*^

Treatments	7 days after application		14 days after application	
	Disease index	Control efficacy (%)	Disease index	Control efficacy (%)
A	3.61 ± 0.21b	43 ± 3.23c	5.86 ± 0.65b	65 ± 3.55b
B	2.70 ± 0.20c	57 ± 3.58b	4.78 ± 0.58b	72 ± 3.08ab
C	2.46 ± 0.23c	61 ± 3.53ab	4.09 ± 0.42b	76 ± 2.62a
D	2.06 ± 0.25c	67 ± 3.84a	4.34 ± 0.47b	74 ± 2.97a
CK	6.31 ± 0.27a	/	16.86 ± 0.66a	/

a The microcapsules were applied with the concentration of (A) 1,500 g·(hm^2^)^−1^; (B) 3,000 g·(hm^2^)^−1^; or (C) 7,500 g·(hm^2^)^−1^. (D) The B. subtilis wettable powder was applied with the concentration of 750 g·(hm^2^)^−1^. CK was sprayed with water as an untreated control. The forward slash symbol (/) showed CK has no control efficacy compared with different treatment. Different lowercase letters in the same column indicate significant difference between treatments by Duncan's multiple-comparison test (*P *< 0.05). Data are mean ± SE (*n *= 3).

## DISCUSSION

The effectiveness of field biocontrol agents is highly dependent on climatic conditions and is unstable under field conditions (20). Microcapsule embedding technology has been shown to improve the survival of biocontrol bacterium, which inhibited pest and plant pathogens in their desired environment (21). In the present study, *T. asperellum* was successfully encapsulated in sodium alginate microcapsules, which slowed down the release of the conidia and improved their biocontrol efficiency toward cucumber powdery mildew. In field trials, the control efficiency of alginate monolayer microcapsules on powdery mildew was 76% (Table 4). Saberi-Riseh et al. (22) reported that Streptomyces fulvissimus microcapsules not only significantly increased plant growth, but also showed a control efficiency of 90%. Compared with free bacteria, the application of nanoformulations prepared with P. fluorescens VUPF5 and T17-4 strains on potato plants under greenhouse conditions significantly reduced the Fusarium solani growth and presented efficient potato dry rot control (23). A greenhouse experiment showed that the disease severity of plants treated with alginate and whey protein or peanut butter concentrate was reduced by 40 to 90% compared with the control plants treated with P. fluorescens VUPF506 (24). Therefore, microencapsulation of microorganisms provides a possible solution to overcome their instability under adverse environments.

In this study, the SA microcapsules produced using the extrusion method and containing conidia of the *T. asperellum* were characterized. The SA microcapsules had a median diameter of 151.8 μm, showed a spherical morphology, and were able to encapsulate the conidia. Extrusion is an easy, mild, and inexpensive method to prepare microcapsules, which reduces the core material damage and maintains the activity of the core material with high encapsulation efficiency (25). Microencapsulated Lactococcus lactis ABRIINW-N19 was prepared by mixing alginate with fructooligosaccharides and inulin using the extrusion method, resulting in a high encapsulation efficiency (≥98.4%) (26). Lactobacillus acidophilus was encapsulated using alginate as encapsulation material, with 98% maximum encapsulation efficiency (27). A high encapsulation yield (72.5%) was obtained for L. reuteri CRL 1324 cells encapsulated with xanthan-gellan material produced by the extrusion method (28). These studies showed that microcapsules comprising SA presented high encapsulation efficiencies, likely because the encapsulation material formed a protective shell around the conidia.

Microencapsulation is used to control the release of encapsulated cells. Release is an important indicator that reflects the sustained release effect and durability of microcapsules (29). There are two stages in the release process: rapid release and slow release (30). The water content and swelling rate are related to microcapsule release (31). In this study, initially, the microcapsules released the conidia rapidly, but then the release slowed after the first week, representing the two stages of release from microcapsules, which met the requirements for sustained release of the conidia from the microcapsules. This can be explained by the lower stability of the SA microcapsules, leading to their degradation, as shown by scanning electron microscopy (SEM) analysis. The SEM images showed many cracks and pores on the surface. Therefore, the microcapsules could encapsulate microorganisms and release them in a sustained manner.

Another important factor that affects *T. asperellum* biocontrol is UV irradiation. Microcapsules can protect microorganisms from the effects of soil moisture and pH, and UV in terms of microbial survival and longevity when delivered by foliar spraying (22, 32). In this study, the survival rate of the microencapsulated *T. asperellum* conidia was about 50% after a UV irradiation for 0.5 h, while the survival rate of free conidia was 27%. Khorramvatan et al. (33) encapsulated B. thuringiensis subsp. *kurstaki* and then exposed them to UV light for 7 days. The spore viability and mortality toward *Ephestia kuehniella* larvae were 68% and 85%, respectively, whereas the spore viability and larval mortality of the free spore formulation were 40% and 50%, respectively. Previous research has shown that the insecticidal activity against Spodoptera exigua larvae of nonmicroencapsulated baculovirus was reduced by 27.27% under UV treatment, whereas that of the microencapsulated baculovirus was only reduced by 8.89% (34). In conclusion, microencapsulation of biological control products results in significantly improved stability and viability of conidia even under UV irradiation.

Storage stability directly determines the shelf life of the conidia. For living conidia, low temperature contributes to conidia activity. Thus, microcapsules might be a good strategy to overcome the challenge of higher temperature storage. In this study, the microcapsules retained high activity at 90 d under two storage temperatures (25°C and 4°C). The encapsulated conidia presented better viability at both storage temperatures compared with bare conidia. The survival rate of the conidia in the microcapsules was 23% compared with 3% for the nonencapsulated conidia at 54°C for 15 d. Similarly, He et al. (35) showed that the stability of B. thuringiensis crystal protein in free crystals was worse than that of microencapsulated crystals at a high temperature (50°C). Setiarto et al. (36) reported that microcapsules encapsulated with modified taro starch incubated in a water bath at 50°C, 60°C, 70°C, and 80°C for 30 min provided the best heat resistance and protection for *L. plantarum* SU-LS36. Thus, microcapsules were able to maintain the viability of conidia.

*Trichoderma* spp. are universal and widely used biocontrol agents (37). The number of *Trichoderma* spp. agents found internationally has increased exponentially, with more than 250 products registered, and these agents are widely used in leaf spraying and soil treatment (38–40). Under field conditions, *T. asperellum* microcapsules showed high control efficacy against cucumber powdery mildew. Previous research showed that *T. harzianum* and *T. viride* exhibited strong inhibitory activities against *S. fuliginea*, with 44.07% and 39.72% control efficiency, respectively (41). *T. harzianum* suppressed strawberry powdery mildew damping-off effectively (42). *T. harzianum* T39 spray reduced the severity of powdery mildew by 97%; however, as the epidemic progressed, its effectiveness dropped to 18 to 55% (43). In contrast, the control efficacy of *T. asperellum* microcapsules increased as the infection progressed in this study, which could probably be attributed to the protection provided by the SA and slow conidia release from the microcapsules. In this study, *T. asperellum* microcapsules at a concentration of 7,500 g·(hm^2^)^−1^ were as effective as B. subtilis wettable powder against cucumber powdery mildew, at a 10-times reduced dosage compared with B. subtilis wettable powder.

### Conclusions.

In the present study, *T. asperellum* conidia were used as the model for microencapsulation preparation. *T. asperellum* conidia were effectively encapsulated by 1% SA, 4% CaCl_2_, curing for 15 min, and 2:1 (wt/wt) of conidia to SA. The viability of the encapsulated conidia was enhanced compared with that of bare conidia. Moreover, at room temperature and 4°C, 38% and 51%, respectively, of the original *T. asperellum* conidia activity were obtained after storage for longer than 3 months, while only 23% and 35%, respectively, of the original activity remained for the nonencapsulated conidia. The survival rate after heat storage for 15 days and UV treatment for 0.5 h were 23% and 50%, respectively, which were significantly higher than the 3% and 27%, respectively, of the nonencapsulated conidia. Therefore, the encapsulated *T. asperellum* conidia were stable under various environmental stresses. In field trials, the microcapsules showed higher control efficiency than the bare conidia toward cucumber powdery mildew. These results suggested that the *T. asperellum* microcapsules are practical and environmentally friendly microbial agents for the control of cucumber powdery mildew.

## MATERIALS AND METHODS

### Fungal strain.

*T. asperellum* CB1 (GenBank: JX270638.1) was isolated and deposited in the Laboratory of Forest Microbiology, Northeast Forestry University, China. *T. asperellum* was grown on potato dextrose agar (PDA) medium (200 g of potato, 20 g of dextrose, 15 g of agar, and 1,000 mL of distilled water) at 25°C for 5 d. Then, 5 mL of sterile water was added to each petri dish to collect the conidia. The suspension in sterile distilled water was adjusted to a concentration of 10^9^ conidia mL^−1^ to prepare the microcapsules.

### Biocompatibility analysis of SA.

SA was added into PDA medium to reach final concentrations of 0%, 0.5%, 1.0%, 1.5%, and 2% (wt/vol), followed by sterilization at 121°C for 20 min. A 5 mm mycelium disc was cut from an active growing colony using a 5 mm diameter puncher and inoculated into the center of the PDA plates containing different concentrations of SA. A 5-mm mycelial disc cut from the active growing colonies was inoculated into the center of a PDA plate without SA as a control. Each treatment was performed in three replicates, and the diameter of the mycelium colonies was measured every 24 h for 7 days. The mycelium growth rate was calculated using the following formula: Growth rate (mm/d) = Diameter of mycelium colony −5 (mm)/Number of growth days (d), where 5 mm is the diameter of the incubation strain mycelial discs.

For biocompatibility screening, SA (0.5%, 1.0%, 1.5%, and 2% [wt/vol]) was added to PDA medium plates. A 50-μL aliquot of conidia solutions was uniformly spread on the mixed PDA medium containing SA in each plate, and a PDA medium without SA was used as a control. Each medium was treated with conidia solution for three replicates and incubated at 25 ± 2°C for 48 h, after which the number of fungal colonies was counted.

### Preparation of microcapsules.

**(i) Materials.** SA (100 to 200 mPa.s of low viscosity) and CaCl_2_ were purchased from Sinopharm Chemical Reagent Co., Ltd. (Shanghai, China). All other chemicals were of analytical grade and used as received without further purification.

**(ii) Preparation of alginate monolayer microcapsules.** The *T. asperellum* conidia was mixed with different concentrations of sterile SA solution (0.5%, 0.8%, 1%, 1.5%, and 2% [wt/vol]) at a ratio of 1:1 (wt/wt). The mixture was dropped through a nozzle of 200 μm into a 2% CaCl_2_ solution with a 60% stirring speed for continuous agitation, using a syringe pump with a 50 mL disposable syringe employing the coextrusion laminar jet break-up technique (BUCHI Encapsulator B-395 Pro, Basel, Switzerland). Pliable microbeads were cured for 10 min in CaCl_2_ solution at room temperature, washed three times with sterile water, and finally filtered to obtain SA monolayer microcapsules. The microcapsules were stored at 4°C until further use and were also allowed to air dry at room temperature to reach their equilibrium moisture content. The encapsulation efficiency was used to evaluate the encapsulation effect of the microcapsules (12). Encapsulation efficiency was calculated using equation and the measurements were replicated three times (44). EE (%) = (N_t_ − N_f_)/N_t_ × 100, where N_t_ is a total number of spores in the sample, N_f_ is the number of spores in the filtrate, and the conidial concentrations were quantified using a hemocytometer.

The CaCl_2_ concentrations, the ratio of conidia to SA, the stirring speed, and the curing time of microcapsules were optimized separately to investigate their effects. When optimizing the CaCl_2_ concentrations, the conidial powder was mixed with 1% SA at a ratio of 1:1 (wt/wt) and then injected into different CaCl_2_ solutions (1%, 2%, 3%, 4%, and 5% [wt/vol]) with a 60% stirring speed for 10 min and cured for 10 min in CaCl_2_ solution at room temperature. When optimizing the ratio of conidia to SA, 1% SA was mixed with conidia at different ratios (3:1, 2:1, 1:1, 1:2, and 1:3 [wt/wt]), injected into 2% CaCl_2_ solutions with a 60% stirring speed for 10 min, and cured for 10 min in CaCl_2_ solution. When optimizing the stirring speed, different stirring speeds (20%, 40%, 60%, 80%, and 100%) during microcapsule preparation were selected. One percent SA, a ratio of 1:1 (wt/wt) conidia to sodium alginate, 2% CaCl_2_ and curing for 10 min were maintained. When optimizing the curing time, 1% SA was mixed with conidia at a ratio of 1:1 (wt/wt), then injected into 2% CaCl_2_ solutions with a 60% stirring speed for 10 min, and then curing for 0 min, 10 min, 20 min, 30 min, and 40 min in CaCl_2_ solution at room temperature was carried out. Microcapsules cured for 0 min were directly collected curing approximately 40 s after stirring for 10 min. The SA monolayer microcapsules were obtained by washing three times with sterile water and then filtering through a medium-grade qualitative filter paper (Fuyang beimu filter paper factory, Hangzhou, China. Paper diameter: 12.5 cm; pore size: 15 μm). The encapsulation efficiency and the number of spores embedded in microcapsules were determined as described above.

**(iii) Orthogonal optimization of formulation.** Based on single factor investigation, the L_9_ (3^4^) orthogonal test adopted variance analysis to determine the interaction between the basic factors and minor factors (Table 5). For the orthogonal design, the SA concentrations (factor A), ratio of conidia to SA (factor B), CaCl_2_ concentrations (factor C), and curing time (factor D) were optimized. The encapsulation efficiency of microcapsules was chosen as evaluation indexes.

**TABLE 5:** Factors and levels of different treatment

Level	Factors			
	SA concentrations (wt/vol) (A)	Ratio of conidia to SA (wt/wt) (B)	CaCl_2_ concentrations (wt/vol) (C)	Curing time (min) (D)
1	0.5%	3:1	2%	5
2	0.8%	2:1	3%	10
3	1.0%	1:1	4%	15

### Characterization of microcapsules.

The 2:1 (wt/wt) ratio of conidia to 1% SA was solidified in 4% CaCl_2_ for 15 min at 60% stirring speed as the *T. asperellum* microcapsules. The characterization of the microcapsules were determined after air-drying at room temperature.

**(i) SEM observation.** The morphology and surface topography of the microcapsules was observed using a scanning electron microscope (Apreo C, Thermo Scientific, Waltham, MA, USA).

**(ii) Particle size analysis.** The particle size of the microcapsule powder was determined using a laser particle size analyzer (BT-9300H, Dandong Bettersize Instruments Ltd., Dandong, China). The particle size was represented by D10, D50, and D90 (D10, D50, and D90 represent the particle size with cumulative particle distribution of 10%, median particle size, and cumulative particle distribution of 90%). The samples were measured three times and then the average particle size was calculated.

**(iii) Loading capacity.** First, 100 mg of microcapsules was suspended in 5 mL of 0.2 M NaHCO_3_ and 0.06 M Na_3_C_6_H_5_O_7_·2H_2_O and stirred for 1.5 h to achieve complete dissolution. The conidial concentration was determined using a hemocytometer (RCT basic, IKA, Staufen, Germany). Loading capacity (LC) was expressed as the number of spores (NS) per gram of dry microcapsules and was calculated by the following equation (45): LC = (C_s_ × V_s_)/M, where C_s_ is the conidia concentration in the sample, V_s_ is the volume of the sample, and M_c_ is the weight of the microcapsules. The measurements were replicated three times.

**(iv) Water content.** The microcapsules were weighed as W_0_ and were then fully dried for 5 h at 105°C to a constant weight in a drying oven, and weighed as W_d_. The water content was calculated using the following formula (36): WC (%) = (W_0_ − W_d_)/W_0_ × 100.

**(v) Swelling measurements.** Dried microcapsules (weight recorded as W_d_) were soaked in 10 mL of deionized water for 24 h, the surface water of the microcapsules was removed with filter paper, and then microcapsules were immediately weighted as W_w_. The swelling rate was calculated using the following formula (46): Swelling (%) = (W_w_ − W_d_)/W_d_ × 100.

**(vi) Microcapsule release rate.** To study the release rate of the encapsulated conidia, 0.5 g of the dried microcapsules were immersed in deionized water. The number of conidia released into the solution was counted under an optical microscope after 1, 2, 3, 6, 9, 12, and 15 days.

**(vii) UV stability.** The UV stability of encapsulated and nonencapsulated samples was determined in a UV irradiation chamber. The samples were placed on a plate and then exposed to a UV lamp (30 W) emitting UV rays from a 50 cm distance for 30 min. The survival rate was expressed as a percentage of surviving conidia in the sample after UV irradiation relative to the amount of conidia in the sample before UV irradiation. All tests were performed in triplicate.

**(viii) Storage stability.** The stability of the microcapsules dispersed in different environments was evaluated using the conidia survival rate. To determine their thermal stability, the microcapsules were stored in an oven at 54°C for 15 d, then cooled to room temperature before measuring their survival every 3 d.

To determine their storage stability, the microcapsules and nonencapsulated conidial powder as a control were stored in the dark at 25°C and 4°C for 3 months, respectively. After the treatments, the number of living conidia in each sample was determined every month.

### Control effects of microcapsules on cucumber powdery mildew.

The field trials were carried out at a greenhouse in Enshi city, Hubei province, where cucumber powdery mildew was infested naturally. The field trials were blocked in greenhouse to prevent effects of biotic or abiotic factors. To confirm their efficacy under field conditions, the air-dried microcapsules were sprayed on cucumber. Two field experiments were conducted on 24 November 2021 and 1 December 2021. The *T. asperellum* microcapsules (1.3 × 10^9^ CFU/g) were applied at concentrations of 1,500 g·(hm^2^)^−1^ (A), 3,000 g·(hm^2^)^−1^ (B), and 7,500 g·(hm^2^)^−1^ (C). B. subtilis wettable powder (10^11^ CFU/g) was used as the control at a concentration of 750 g·(hm^2^)^−1^ (D), and an untreated control (CK) was sprayed with water. A total of five treatments and four replicates for each treatment were conducted. In the initial stage of cucumber powdery mildew, cucumber leaves were sprayed evenly with different test formulations at 750 L per hectare. The control efficacy and disease index were investigated on the 7th and 14th day after the second application. Four experimental sites were randomly selected from each plot, and all leaves of two cucumber plants were investigated at each site. The disease index was determined using the criteria shown in Table 6 (47). The disease index and control efficacy were recorded as follows: Disease index = ∑ (Disease grading × Number of leaves at this scale) × 100/(9 × Total number of leaves investigated). Control efficacy (%) = (Disease index of untreated control − Disease index of treatment)/Disease index of untreated control × 100.

**TABLE 6:** The grading criteria for disease index

Disease grading	Symptoms
0	No disease symptoms
1	Less than 5% of the surface of the leaf is infected
3	6%–10% of the surface of the leaf is infected
5	11%–25% of the surface of the leaf is infected
7	26%–50% of the surface of the leaf is infected
9	More than 50% of the surface of the record is infected

### Statistical analysis.

The SPSS 24.0 software (IBM Corp., Armonk, NY, USA) was used for statistical analyses. Significant differences between treatments were assessed using one-way analysis of variance (ANOVA) followed by using Duncan's multiple range test at the *P < *0.05 level.
